# Discrimination of *Camellia* cultivars using iD-NA analysis

**DOI:** 10.1038/s41598-023-44404-z

**Published:** 2023-10-17

**Authors:** Hitomi S. Kikkawa, Mitsuhiko P. Sato, Ayumi Matsuo, Takanori Sasaki, Yoshihisa Suyama, Kouichiro Tsuge

**Affiliations:** 1 National Research Institute of Police Science, 6-3-1 Kashiwanoha, Kashiwa, Chiba 277-0882 Japan; 2 Kazusa DNA Research Institute, 2-6-7 Kazusakamatari, Kisarazu, Chiba 292-0818 Japan; 3 Kawatabi Field Science Center, Graduate School of Agricultural Science, Tohoku University, 232-3 Yomogida, Naruko-Onsen, Osaki, Miyagi 989-6711 Japan

**Keywords:** Biological techniques, Plant sciences

## Abstract

Recently, many new cultivars have been taken abroad illegally, which is now considered an international issue. Botanical evidence found at a crime scene provides valuable information about the origin of the sample. However, botanical resources for forensic evidence remain underutilized because molecular markers, such as microsatellites, are not available without a limited set of species. Multiplexed intersimple sequence repeat (ISSR) genotyping by sequencing (MIG-seq) and its analysis method, identification of not applicable (iD-NA), have been used to determine several genome-wide genetic markers, making them applicable to all plant species, including those with limited available genetic information. *Camellia* cultivars are popular worldwide and are often planted in many gardens and bred to make new cultivars. In this study, we aimed to analyze *Camellia* cultivars/species through MIG-seq. MIG-seq could discriminate similar samples, such as bud mutants and closely related samples that could not be distinguished based on morphological features. This discrimination was consistent with that of a previous study that classified cultivars based on short tandem repeat (STR) markers, indicating that MIG-seq has the same or higher discrimination ability as STR markers. Furthermore, we observed unknown phylogenetic relationships. Because MIG-seq can be applied to unlimited species and low-quality DNA, it may be useful in various scientific fields.

## Introduction

Plants have been commonly utilized as foods, drugs, and gardening by people for life. Therefore, there is an increasing need to identify the origin of botanical samples. Many cultivars of garden plants and crops are produced by plant breeding, and new varieties have been registered. The breeders also have the right to grow and sell the variety exclusively. They can demand to discontinue infringement, which is punishable under Japanese law^1,2^. Many new cultivars have been taken abroad illegally, which has become an international issue^3–5^. Many illegal drugs derived from plants such as *Cannabis sativa* L.^6–11^, *Papaver somniferum*^12,13^, and *Panaeolus cambodginiensis*^14^ are often problematic. Moreover, some toxic plants show strong morphological similarities to edible plants or herbs, and poisoning is frequently caused by accidental ingestion^15–20^. If all toxic plants can be analyzed in detail, we can determine the origin of the sample. In addition, if small plant fragments, such as leaf fragments, found on a suspect are proven to originate from a crime scene, they can serve as evidences linking the suspect to the scene^21–25^. By comparing botanical evidence found at the crime scene with a sample taken from the suspect, we can determine whether the evidence is associated with the suspect.

Discrimination of possible sources among samples or related cultivars should be done using molecular markers, such as microsatellites or single nucleotide polymorphisms (SNPs), because they ideally provide resolution at the genotype level^26^. Many genotyping methods using molecular markers have been developed for plant species, especially for commonly used crops. However, most of them are only capable of distinguishing the differences within a limited set of closely related species. The method is required to discriminate at a higher resolution and should be used for many plant species for novel criminal investigations, such as the protection of breeder’s rights and tracing of the origin of the samples.

**Figure 1:**
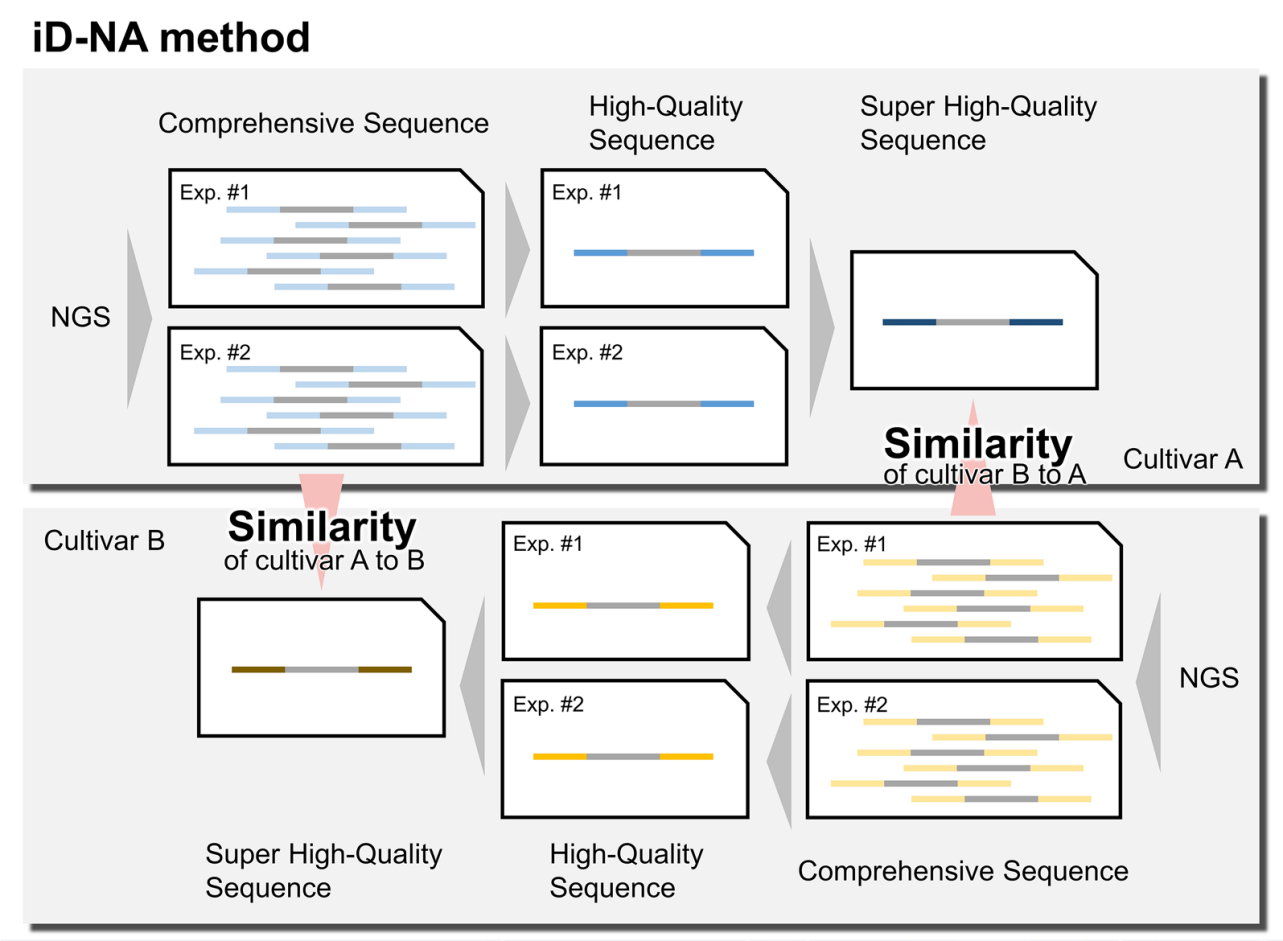
Schematic diagram of the discrimination procedure applied for identification using the iD-NA method. This method directly compares NGS reads and determines the exact matching rate between the target and query samples through three steps: (1) applying stringent filters to NGS raw data to refine sequencing reads, (2) identifying the sequencing reads that should be present in the target sample as reference, and (3) conducting a comprehensive search for sequencing reads that exactly match the target sample.

Recently, next-generation sequencing (NGS) technology has allowed the effective investigation of genome-wide genetic markers^27–31^. MIG-seq is one such technique; it amplifies intersimple sequence repeats (ISSRs), enabling analysis of a large number of anonymous genome-wide regions without prior genetic information. Thousands of regions are amplified from a wide variety of genomes, which effectively represent a reduced genome library. MIG-seq is widely applicable to field samples even with low-quality DNA and/or small quantities of DNA^29^ and can be used for many nonmodel species and those that lack genetic information, including not only plants but also animals and fungi. It can be used in marker-assisted genetic studies, such as ecological, evolutionary, phylogeographic, and genetic mapping studies. NGS using MIG-seq can determine the phylogenetic relationship and population structure, along with cultivar identification and discrimination, which are not possible using previously reported sequencing techniques^32,33^. Furthermore, a novel analysis method called identification of not applicable (iD-NA), which uses MIG-seq results, was reported recently^34^. Unlike widely used programs that use complex processes and occasionally make errors when identifying SNPs (Fig. 1), iD-NA directly compares NGS reads to determine the exact matching rate between the target and query samples. These distinct characteristics indicate the potential of iD-NA for distinguishing among samples, including illegally used plant cultivars.

**Figure 2:**
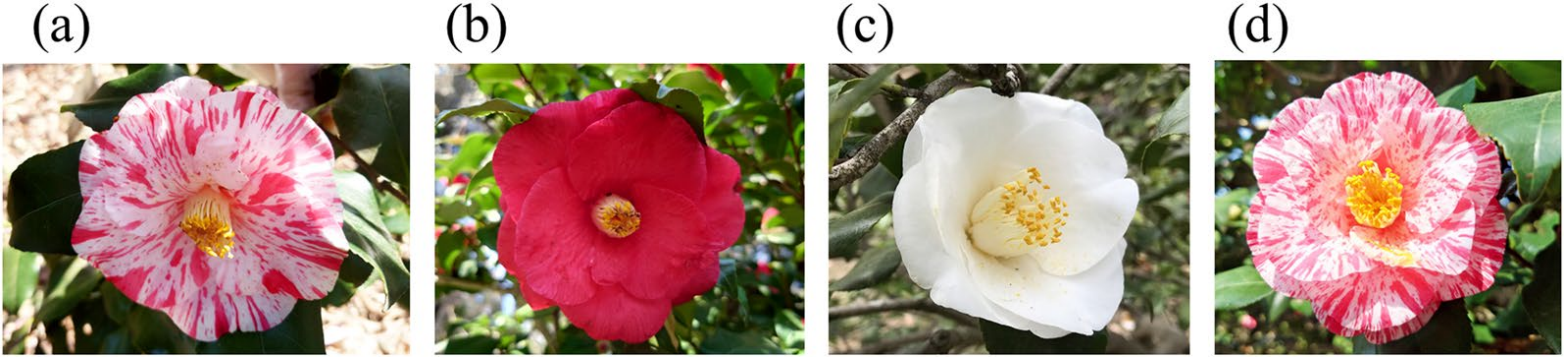
Flower color of “Ezonishiki” and related cultivars. (**a**) “Ezonishiki (original)”; (**b**) “Akaezo (red petal mutant of “Ezonishiki”)”; (**c**) “Shiroezo (white-petal mutant of “Ezonishiki”)”; (**d**) “Ezoshibori”.

The genus *Camellia* contains more than 200 species and is mainly distributed in the southern and southwestern parts of China^35^. The ornamental *Camellia* garden plant has gained substantial popularity as a rare winter-blooming evergreen tree grown in temperate areas. Therefore, the presence of *Camellia* garden plants can serve as an evidence in criminal investigations, indicating the need for discerning their origin. The overwhelming majority of garden *Camellia* cultivars are derived from *Camellia japonica* L. Moreover, *C. japonica* subsp. rusticana (Honda) Kitamura, *C. sasanqua* Thunb., and *C. reticulata* Lindl. have also been utilized, which are used to develop hybrids of the majority of modern *Camellia* cultivars^35^. Many cultivars have origins that are not clearly determined because they have been planted for hundreds of years. It is believed that over 100 named cultivars existed in Japan in the seventeenth century. During the seventeenth and nineteenth centuries, they were introduced into Europe, and many cultivars have been currently bred not only in Japan but also in Europe. In the past several decades, the breeding of interspecific hybrids of the genus *Camellia* has resulted in the production of many diverse new cultivars. New genetic resources have been used to increase diversity among *Camellia* cultivars, and interspecific hybrid cultivars have been more consistently bred than ever before^36^. In cases where new cultivars have been unlawfully taken abroad, it becomes necessary to distinguish between the confiscated samples and original cultivar for court proceedings. Furthermore, elucidating the genealogical relationships can aid in the efficient breeding of new cultivars. However, only a few studies have been conducted on *Camellia* garden plants^37–39^, and the relationships among these plants have not yet been determined. In addition, intracultivar diversity has not been reported. For garden cultivated plants, cutting and seeding are often practiced to increase their number^40^. Therefore, genetic diversity within a cultivar is not very high. Moreover, *Camellia* has many cultivars derived from bud mutation in spite of the variety of traits. For example, “Akaezo (red Ezonishiki)”, “Shiroezo (white Ezonishiki)”, and “Ezonishiki” are all bud mutants (Fig. 2). “Ezonishiki” has a variegated red–white flower, whereas “Akaezo” has a red flower and “Shiroezo” has a white flower.

In this study, we used MIG-seq to analyze cultivars/species within the genus *Camellia*, given the potential for discrimination among *Camellia* garden plants. MIG-seq could discriminate similar samples, such as bud mutations and closely related cultivars, although the samples had a possibility of inbreeding. Cultivars that were classified based on short tandem repeat (STR) markers in a previous study could also be discriminated clearly using MIG-seq, indicating that MIG-seq has the same (or even higher) discriminatory ability as STR markers. Furthermore, we identified unknown phylogenetic relationships among the cultivars. Because MIG-seq has no limitations with regard to adaptable species, MIG-seq may be useful for criminal investigations.

†This study is based on research first reported in the following reference: “Discrimination of genus Camellia using MIG-seq analysis” (in Japanese) (DNA Polymorphism, Volume 29, Pages 25–31, 2021).

## Results and discussion

### Phylogenetic analysis of *Camellia* cultivars/species obtained using MIG-seq

**Figure 3:**
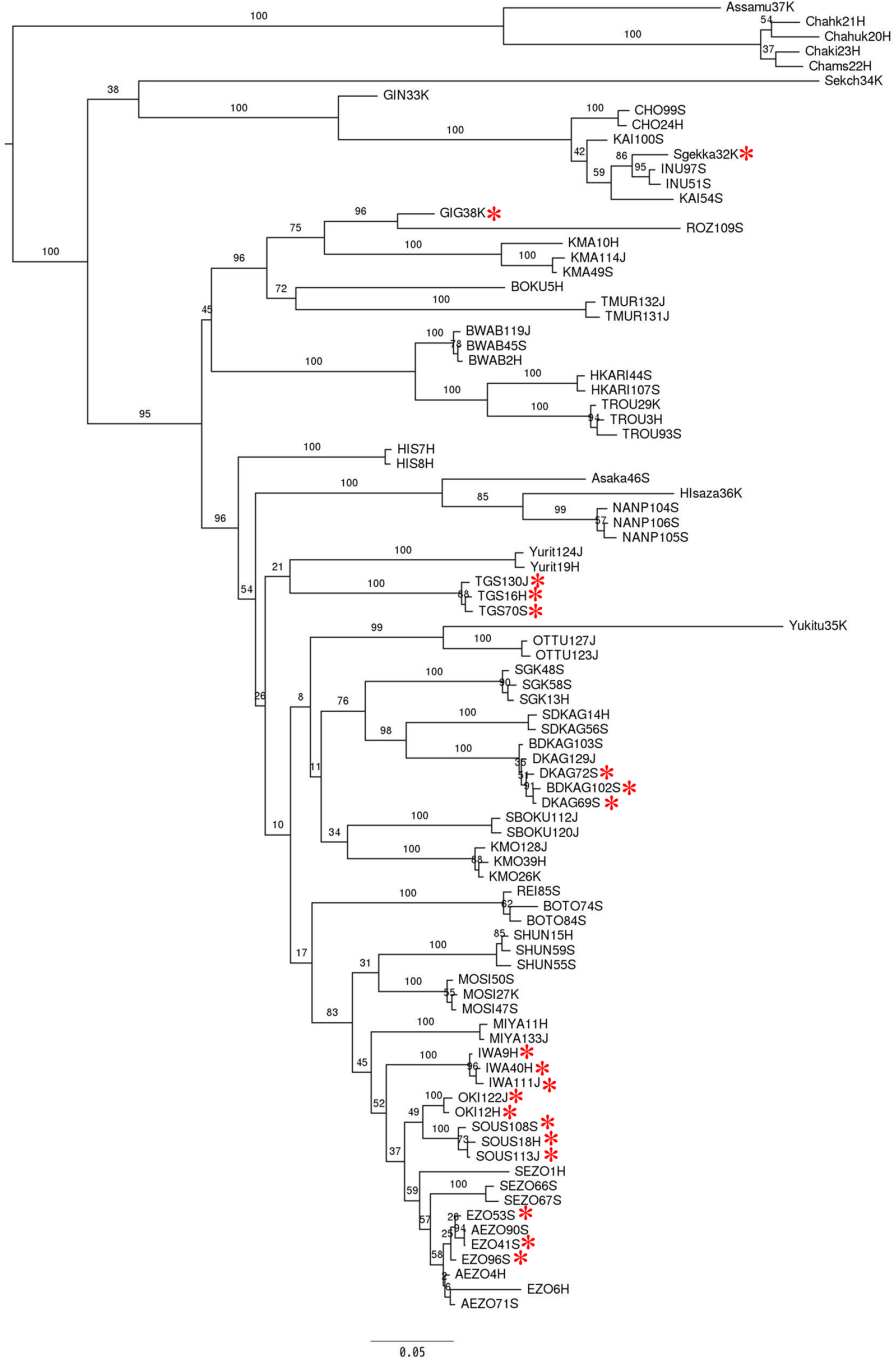
Dendrogram for *Camellia* cultivars generated via MIG-seq analysis. * indicates flowers with a spotted pattern of red and white. The numbers at the branches are bootstrap values.

After filtering the raw reads data, 108,699–322,971 reads per sample were detected. After calling and filtering the SNPs, the reads were grouped into 28,999 loci, and 36,260 variant sites were obtained across the samples. To identify the relationship among 48 *Camellia* cultivars/species, phylogenetic trees were constructed using SNP data (Fig. 3). The same cultivars were clustered together even if they were collected from a different place, indicating that the sampled cultivars were pure strain without intercrossing and hybridization. Some bud mutants belonged to the same clade of their parents’ cultivars. For instance, “Benidaikagura (BDKAG; red Daikagura)” was clustered with “Daikagura (DKAG)”.

Research on phylogenetic trees has revealed the relationships among *Camellia* cultivars that were previously unknown. For instance, “Daikagura(DKAG72S)”, “Ezonishiki (EZO41S, 53S and 96S)”, “Gigantea (GIG38K)”, “Iwaneshibori (IWA9H, 40H and 111J)”, “Okinonami (OKI12H, 112J)”, “Setsugekka (Sgekka32K)”, “Soshiarai (SOUS18H, 108S and 113J)”, and “Tamagasumi (TGS16H, 70S and 130J)” have flowers with a spotted pattern of red and white, but they are not closely related. “Ezonishiki” has the closest relationship with “Soshiarai”; close relationship with “Iwaneshibori” and “Okinonami”; distinct relationship with “Daikagura”, “Tamagasumi”, and “Gigantea”; and the most distinct relationship with “Setsugekka” among them. It is highly possible that samples that show similar characteristics could be discriminated using MIG-seq analysis. If the suspect sells the plant sample that is newly bred and protected by the breeder’s rights, the analyst can reveal the origin of the suspicious sample. This indicates that MIG-seq is useful for forensic discrimination.

### Comparison of SNPs obtained using MIG-seq among different cultivars

**Figure 4:**
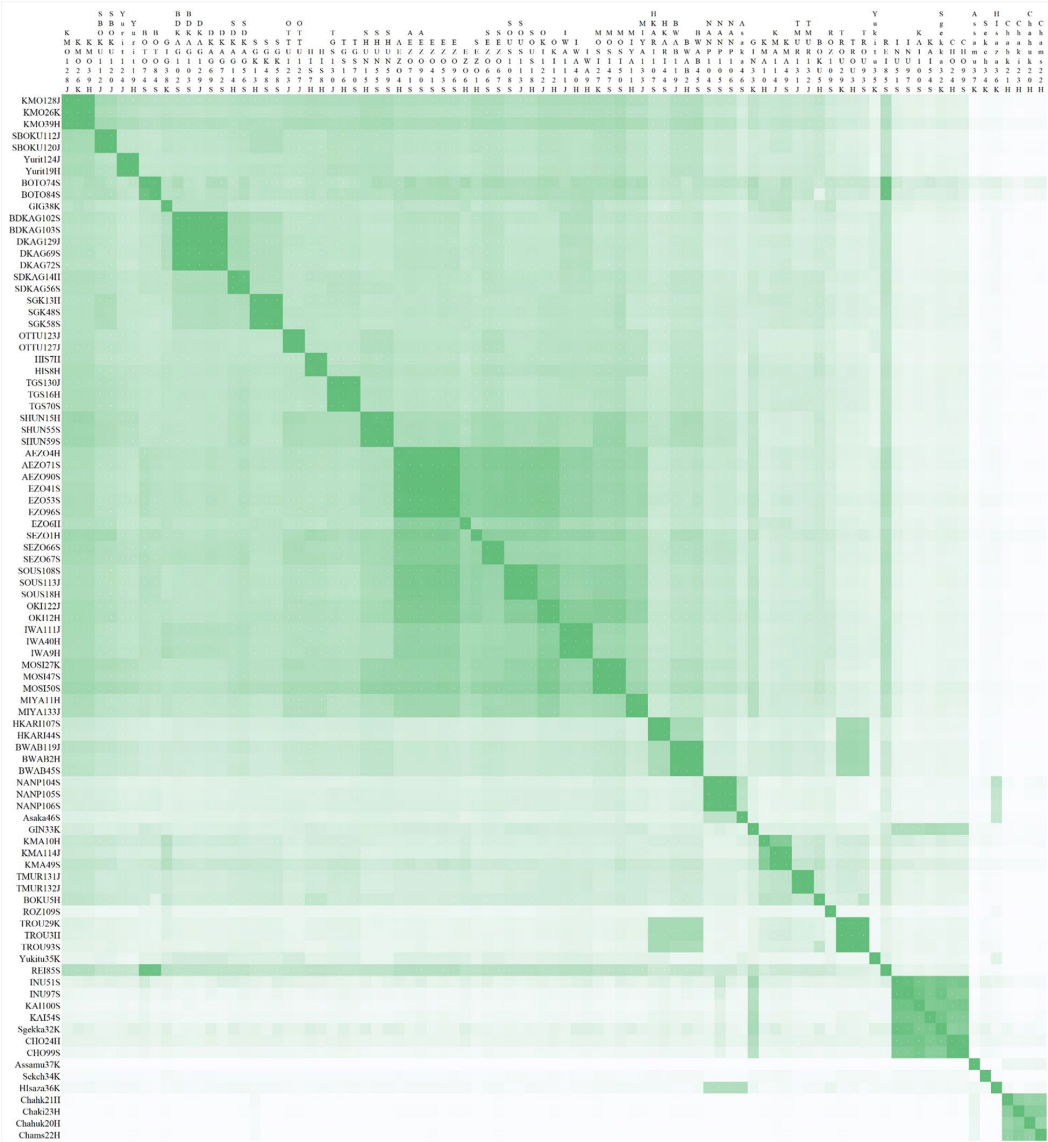
Pairwise comparison of *Camellia* cultivars/species. Histogram showing the shared sequences between two samples. Darker color indicates that more shared sequences were obtained. The sample IDs are shown on the top and left.

To investigate whether MIG-seq can discriminate between cultivars in detail, the output sequences were evaluated using another method called iD-NA. The obtained high-quality sequences of the sample were set to reference data, and the number of reference sequences detected in other cultivars was examined. If a given sample shares all super high-quality sequences of the counterpart, the value of similarity is 1.0. The same cultivars were found to share many sequences even if they were planted in different gardens (Fig. 4 and Table S1), as also evidenced by phylogenetic tree analysis. These results indicate that the iD-NA method is as effective as or more effective than the traditional method of using SNPs and phylogenetic analysis in identifying genetic differences between cultivars.

**Figure 5:**
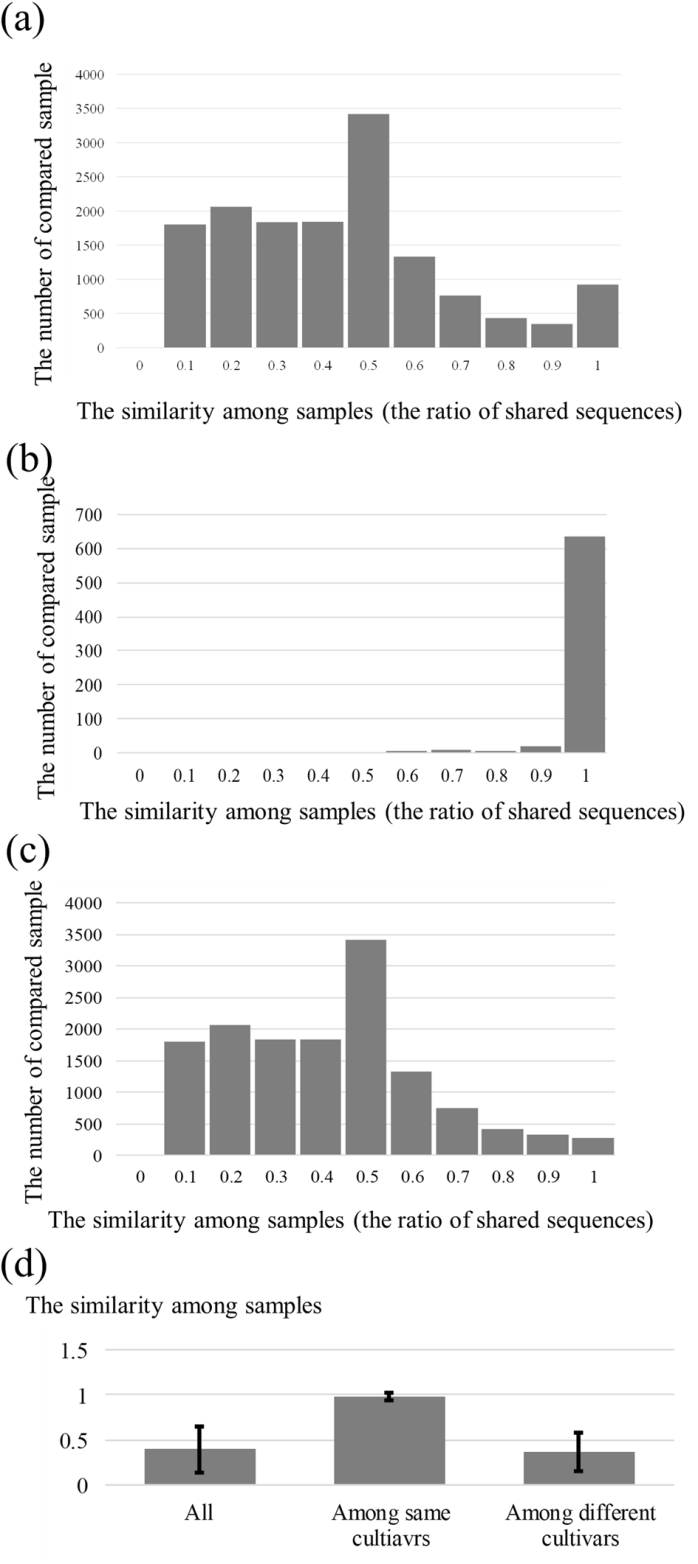
Histogram showing the distribution of the ratio of shared sequences between the two samples. (**a**) Among all samples; (**b**) among the same cultivars; (**c**) among different cultivars; (**d**) average of the samples. If two samples have a closely related phylogenetic relationship and share all high accuracy sequences of the counterpart, the value is 1. The error bar indicates standard deviation.

Next, all samples were compared and validated (Fig. 5). Although the observed heterozygosity was lower than expected, indicating that inbreeding had occurred among the samples in this study (Table S2), the similarity between the same cultivars differed from that between different cultivars. The average of the similarity between the same cultivars was 0.98 ± 0.05 (mean ± s.d.) and the comparison indicated that all super high-quality sequences could be observed in other same cultivar samples. Only three cultivars (KMA10H, SEZO1H and SGK101S) had moderate similarity (Table S1). KMA10H and other same cultivar samples shared a value of 0.74–0.80, which is lower than that of the other “Kumagai (KMA)” samples. KMA has several lines which were bred in different regions of Japan^35^. This raises the possibility that KMA10H has a different line from the other samples. SEZO1H and the other same cultivar samples (SEZO113J and SEZO126J) shared a value of 0.65–0.74. They were sampled from different gardens, indicating that they were derived from different sources. SEZO is a white bud mutant of “Ezonishiki”. To the best of our knowledge, no studies on the mechanisms of bud mutations of *Camellia* species have been conducted. However, previous studies suggested that white-petal varieties were obtained from the colored variety by cosuppressing the expression of genes encoding chalcone synthase and dihydroflavonol-4-reductase^41–43^. These genes are involved in the biosynthesis of anthocyanin, which are a colored class of flavonoids responsible for the pink, red, violet, and blue colors of flowers. It is possible that different factors resulted in white bud mutants, and these different lines were dealt with as a same cultivar because they could not be distinguished based on morphological features. SGK101S and other same cultivars shared a value of 0.60–0.70. It is also likely that SGK101S was derived from different lines of other SGK samples. SGK (“Shiragiku”) is an old cultivar and was recorded in a literature published in 1695^44^. The methods for plant propagation might have changed during the course of hundreds of years. It is also possible that some ancient plant propagation methods might have increased the diversity in the same cultivars. These results suggest that MIG-seq can discriminate not only the same cultivars but also different lines within the same cultivars.

“Gigantea (GIG)”, “Okinonami (OKI)”, “Otometsubaki (OTTU)”, “Setsugekka (Sgekka)”, and “Ginryu (GIN)” could be discriminated using STR markers, as discussed in a previous study^37^. The same cultivars shared a value of 0.99–1 (99–100%), although the different cultivars shared a value of 0.14–0.57 (14–57%; Table 1). This indicates that the iD-NA method using MIG-seq exhibits comparable or superior discrimination ability to STR markers. Moreover, it enables the analysis of species that cannot be studied using intraspecies analysis methods, providing a high-resolution approach using iD-NA with NGS.

**Table 1:** Pairwise comparisons among cultivars discriminated using STR markers, as discussed in a previous study^37^.

Sample	GIG38K	GIN33K	OKI122J	OKI12H	OTTU123J	OTTU127J	Sgekka32K
GIG38K	–	0.36	0.40	0.45	0.36	0.36	0.20
GIN33K	0.26	–	0.29	0.29	0.24	0.25	0.52
OKI122J	0.44	0.44	–	1.00	0.49	0.50	0.15
OKI12H	0.46	0.44	0.99	–	0.49	0.50	0.17
OTTU123J	0.37	0.32	0.45	0.45	–	1.00	0.14
OTTU127J	0.39	0.33	0.45	0.45	1.00	–	0.14
Sgekka32K	0.19	0.57	0.14	0.17	0.17	0.17	–

The obtained high-quality sequences of the sample were set as per reference data and the examination of how many reference sequences were detected in other cultivars was conducted. If a given sample shares all super high-quality sequences of the counterpart, the value of similarity is 1.0 counterpart, the value of similarity is 1.0

### Comparison of SNPs obtained using MIG-seq within the same and related cultivars

**Figure 6:**
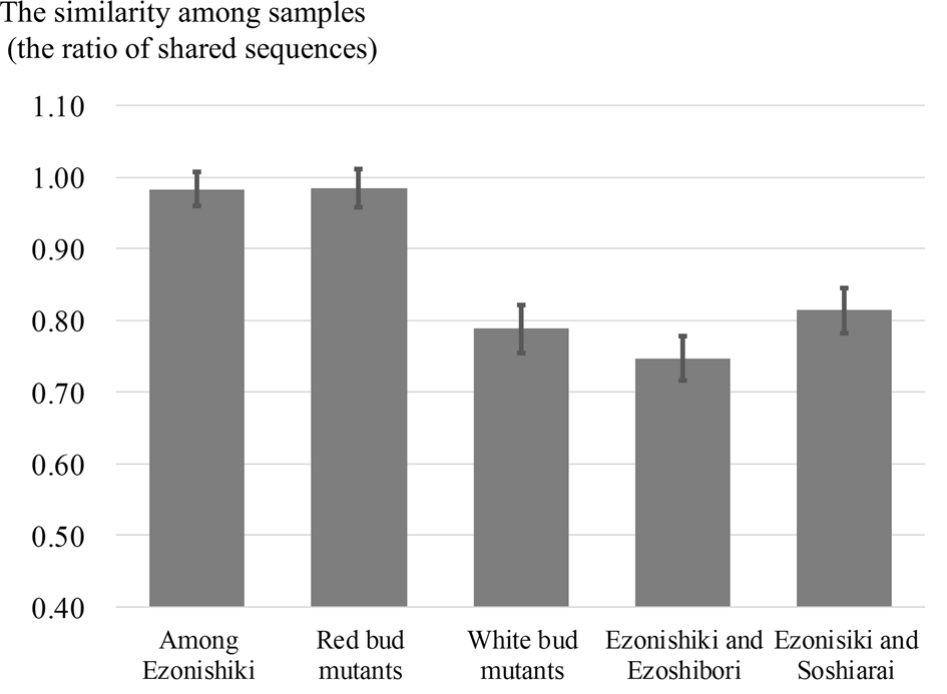
Average of the ratio of the shared sequences among “Ezonishiki” and the related cultivars. If the two samples have a closely related phylogenetic relationship and share all high accuracy sequences of the counterpart, the value is 1. The error bar indicates standard deviation.

We investigated the differences among bud mutations, namely, “Ezonishiki (original)”, “Akaezo (red petal mutant of “Ezonishiki”)”, and “Shiroezo (white-petal mutant of “Ezonishiki”)”. The similarity between the original cultivars and red petal mutants (0.98 ± 0.03) was almost the same as that between the original cultivars (0.98 ± 0.02), whereas more differences were found between the original cultivars and white-petal mutants (0.79 ± 0.03; Figs. 6, S1). This is in good agreement with the explanation by the planted park of “Ezonishiki” and “Ezoshibori” that in “Ezonishiki”, changing to the red bud mutant from the original cultivar is easier than changing to the white bud mutant. It was reported that some genes or transposable elements introduced white color into colored flowers or some color into white flowers, resulting in variegated flowers^45–49^. This variegated flower color is valuable horticulturally because of its beauty, and a variegated flower is therefore treated as the original cultivar horticulturally. However, biologically original cultivars have a single color, such as a red flower. Changing to an absolutely different flower may be more difficult than to a variegated flower. These findings suggest that MIG-seq can discriminate between intracultivar differences.

Significant morphological differences in sepals between “Ezonishiki” and “Ezoshibori (EZO1H)” have not been reported. Both “Ezonishiki” and “Ezoshibori” have variegated red–white flowers (Fig. 2a,d). Moreover, it was explained by the planted park of “Ezoshibori” that “Ezoshibori” is not a common cultivar name, and therefore, “Ezoshibori” may be a synonym of “Ezonishiki”. However, the similarity between “Ezonishiki” and “Ezoshibori” (0.75 ± 0.03) was lower than that of the original “Ezonishiki” samples (0.98 ± 0.02; Figs. 6, S1). Our results suggest that they are not classified under the monophyletic group and that the degree of difference was the same as the white-petal mutant (0.79 ± 0.03). These findings indicate that iD-NA with MIG-seq could discriminate between the closely related groups that could not be distinguished based on morphological features.

To investigate whether iD-NA could discriminate closely related cultivars, we compared “Ezonishiki” and “Soshiarai” based on SNPs via phylogenetic analysis. “Soshiarai” was the most closely related cultivar of “Ezonishiki” (Fig. 3). “Ezonishiki” and “Soshiarai” shared a value of 0.81 ± 0.03, whereas “Ezonishiki” cultivars shared a value of 0.98 ± 0.02 (Figs. 6, S1). These results indicate that iD-NA could discriminate closely related samples as expected. Our method can determine clear differences between the samples according to the genetic distances.

In this study, we analyzed *Camellia* cultivars or species using iD-NA with MIG-seq. We found that iD-NA could discriminate very similar samples, such as bud mutations and closely related samples, although the samples had a possibility of inbreeding. MIG-seq and iD-NA have no limitation of adaptable species. Every species has a potential to become a forensic sample. Although we cannot develop polymorphic markers for every species, iD-NA with MIG-seq can be used to facilitate criminal investigations. Moreover, this method can evaluate unknown samples with regard to biological features, polyploidy, heterogeneity, and mating patterns, such as selfing, apomixis, and vegetative reproduction, and the similarities are easy to compare using simple programs and parameter settings and the differences can be easily visualized and analyzed. For example, a phylogenetic tree is useful for visually understanding the relationship between samples; however, SNPs for the phylogenetic tree obtained using MIG-seq without a reference genome depend on many parameters of SNP calling and filtering based on genetic and ecological mechanisms. If different samples are used, the tree changes. Because the results of iD-NA do not change based on samples, explaining the results in court may be easier than explaining the results of existing complex analyses.

iD-NA may be also applicable for breeding. For example, it is important to clarify the origins of classical cultivars to develop new horticultural varieties. Herein, the unknown relationship among the cultivars was determined and different cultivars could be discriminated. Our results provide new insights into intraspecies analysis for every plant, which will be useful in various scientific fields.

## Materials and methods

### Samples and DNA preparation

We selected *Camellia* cultivars/species that are popular in Japan and often planted in many gardens, assuming that those cultivars have the potential to be forensic samples. Tables S3 and S4 show the target plants. Overall, 122 samples (48 *Camellia* cultivars/species) were used. Specimens were provided by the Koishikawa Botanical Garden (K), Saitama Greenery Promotion Center (H), Jindai Botanical Garden (J), and Musashi-Kyuryo National Government Park (S). Samples were collected with permissions obtained from the parks where they were planted. The sample name indicates the abbreviation of the cultivar name, sample identification number, and abbreviation of the planted park. For example, “KMA10H” indicates “Kumagai” planted in the Saitama Greenery Promotion Center.

Total genomic DNA was extracted from the samples using the DNeasy Plant Mini Kit (Qiagen, Hilden, Germany) in accordance with the manufacturer’s instructions and was stored at − 20 °C until analysis.

### MIG-seq analysis

We constructed a MIG-seq library with two polymerase chain reactions (PCRs). Approximately 1 μL of DNA was used for the first PCR as a template DNA. The first PCR step was performed to amplify ISSR from genomic DNA with MIG-seq primer set-1^29^. The fragments were amplified using the Multiplex PCR Assay Kit Ver. 2 (TaKaRa Bio Inc., Otsu, Shiga, Japan) with 7-μL reaction volumes in a thermal cycler with the following conditions: initial denaturation at 94 °C for 1 min, followed by 25 cycles at 94 °C for 30 s, 38 °C for 1 min, 72 °C for 1 min, and finally 72 °C for 10 min.

The objective of the second PCR was to add complementary sequences that serve as an index for subsequent analyses to the primary PCR products^29^. The fragments were amplified using PrimeSTAR GXL DNA polymerase (TaKaRa Bio Inc., Otsu, Shiga, Japan) with 6-μL reaction volumes in a thermal cycler with the following profile: 12 cycles at 98 °C for 10 s, 54 °C for 15 s, and 68 °C for 1 min. Moreover, the PCR products were selected and cleaned based on library size using AMPure XP (Beckman Coulter, Brea, CA, USA). The sequencing of the multiplexed library was performed using MiSeq Reagent Kit v3 (150 cycle) (Illumina, San Diego, CA, USA) with an Illumina MiSeq Sequencer. The reads including adapter and low-quality sequences were filtered and the first and last nucleotides were trimmed using Trimmomatic^50^. The details of the filtering process are further discussed in the following section about a new cultivar discrimination method.

The filtered reads were then input into Stacks v. 2.59, which is a software that identifies loci, and were used to detect SNPs^51^. The M (maximum distance [in nucleotides] allowed between stacks) and N (maximum distance allowed to align secondary reads to primary stacks) parameter values for the stacks were set to 1 and 1, respectively. In the population module, the minimum number of a population (p) in which a locus must be present was set as 1 and the percentage of individuals within a sample was set to 0.1. Min_maf (minimum minor allele frequency) was set as 0.025 and other parameters were set as default. RAxML v.8.2.12^52^ was used to construct phylogenetic trees. Analysis was performed using a maximum likelihood search for 1000 rapid bootstraps. The phylogenetic tree was visualized using Figtree v1.4.4. (http://tree.bio.ed.ac.uk/software/figtree/). The number of alleles, effective number of alleles, observed heterozygosity, expected heterozygosity, and inbreeding coefficient were calculated using GENODIVE^53^.

We implemented a new cultivar discrimination method called the iD-NA^34^. This method compares NGS reads directly and obtains the exact matching rate between the target and query samples using three steps: (1) stringent filtering of sequencing reads from NGS raw data, (2) identification of sequencing reads that should be present as a reference in the target sample, and (3) comprehensive search for sequencing reads matching exactly with the target sample. In the first step, we recalled nucleotides without any mismatch in barcode sequences from the MiSeq raw data using bcl2fastq v2 (Illumina). It is a stricter setting than the default setting allowing one mismatch. When the base-called quality of barcode was less than quality value (QV) 30, even by a single base, the sequencing reads were removed using in-house script. The reads with adapter sequences or low-quality nucleotides less than QV 30 in the mean of any windows of four nucleotides were removed using Trimmomatic. In the second step, over 10 sequencing reads matching perfectly to the whole length of paired-end reads were obtained as high-quality unique sequencing reads for each sample. The high-quality sequencing reads shared by both of the PCR duplicates were obtained as super high-quality sequencing reads of reference for each cultivar. In the third step, sequencing reads that appeared once in either of the PCR duplicates were obtained as comprehensive sequences for each cultivar. Whole comprehensive sequences of a query cultivar were searched against a super high-quality sequence of a target cultivar, and the exact matching rate to target was calculated as the similarity of the two cultivars using in-house scripts. These searches for within-sample matching in the second step and between-cultivar matching in the third step also included reverse complementary sequences.

### Ethics declarations

The use of plants in this study complied with relevant institutional, national, and international guidelines and legislation.

### Supplementary Information

Supplementary Information.

Supplementary Figure S1.

Supplementary Table S1.

Supplementary Table S2.

Supplementary Table S3.

Supplementary Table S4.

Supplementary Table S5.

## Data Availability

All data generated or analyzed for this study are included in this published paper (and its Supplementary Information files). The datasets generated during the current study are available in the DDBJ Sequence Read Archive repository under accession numbers DRR477561–DRR477682.
