# Supplementary material for: Discrimination of Camellia cultivars using iD-NA analysis
Source: Sci Rep. 2023 Oct 17;13:17674. doi: 10.1038/s41598-023-44404-z (PMC10582245; doi:10.1038/s41598-023-44404-z)
Supplement: Supplementary file 1 — Supplementary Information. [file 41598_2023_44404_MOESM1_ESM.docx]

Figure S1 The pairwise comparison among “Ezonishiki” and related cultivars. Histogram showing the distribution of the ratio of shared sequences (a) among original cultivars; (b) between original cultivars and red petal mutants; (c) between original cultivars and white-petal mutants; (d) between original cultivars and “Ezoshibori”; and (e) between original cultivars and a closely related cultivar (Soushiarai).
