# Supplementary figures and images for: Discrimination of Camellia cultivars using iD-NA analysis
Source: Sci Rep. 2023 Oct 17;13:17674. doi: 10.1038/s41598-023-44404-z (PMC10582245; doi:10.1038/s41598-023-44404-z)

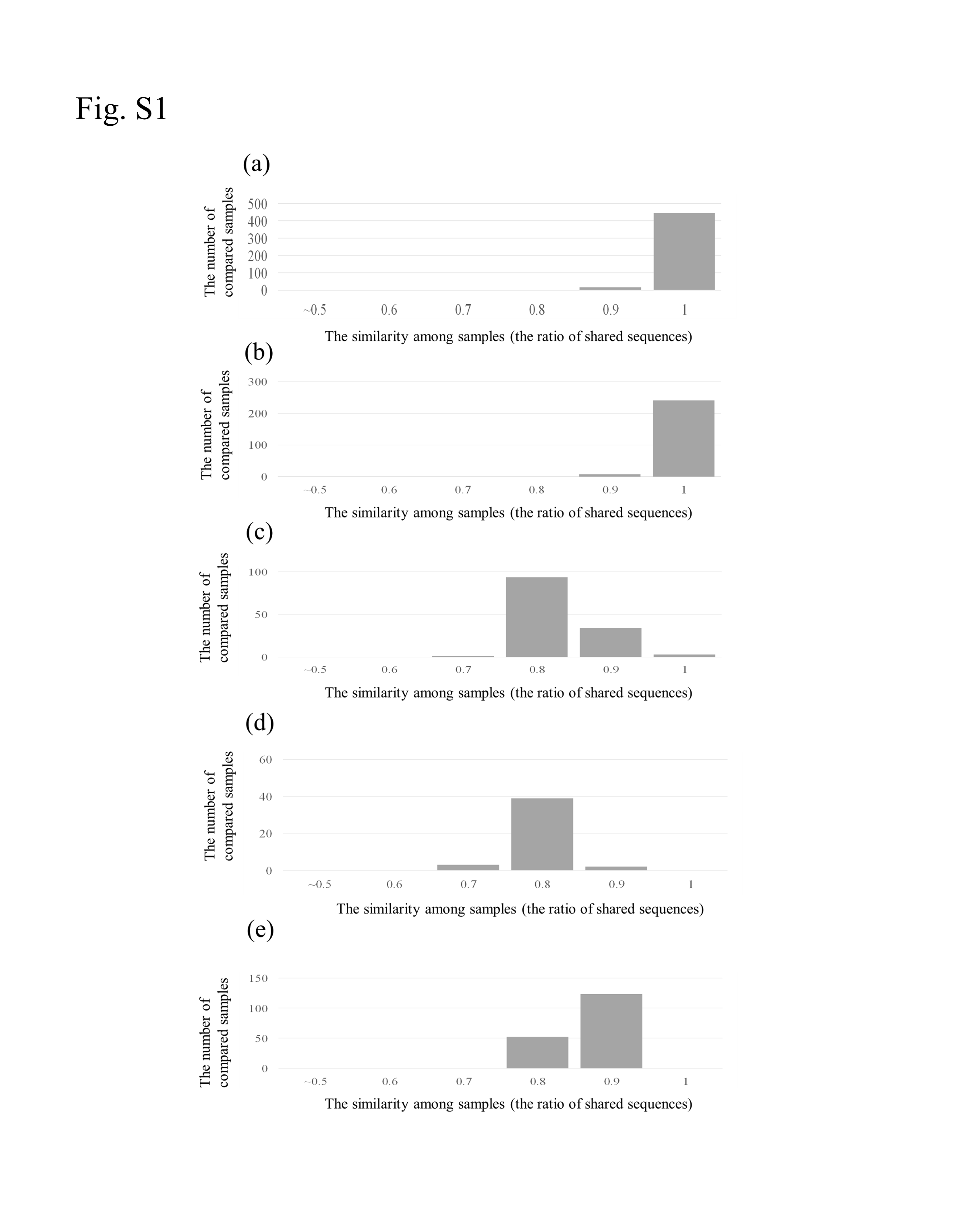

Supplement: Supplementary file 2 — Supplementary Figure S1. [file 41598_2023_44404_MOESM2_ESM.tif]
